# PRIMA-1^MET ^induces nucleolar translocation of Epstein-Barr virus-encoded EBNA-5 protein

**DOI:** 10.1186/1476-4598-8-23

**Published:** 2009-03-26

**Authors:** György Stuber, Emilie Flaberg, Gabor Petranyi, Rita Ötvös, Nina Rökaeus, Elena Kashuba, Klas G Wiman, George Klein, Laszlo Szekely

**Affiliations:** 1 Department of Microbiology, Tumor and Cell Biology (MTC), Karolinska Institute, S-171 77 Stockholm, Sweden; 2 Karolinska Institute Visualization Core Facility (KIVIF), Karolinska Institute, S-171 77 Stockholm, Sweden; 3 Center for Integrative Recognition in the Immune System (IRIS), Karolinska Institute, S-171 77 Stockholm, Sweden; 4 Swedish Center for Disease Control (SMI), S-171 82 Solna, Sweden; 5 Deptarment of Oncology-Pathology, Cancer Center Karolinska (CCK), Karolinska Institute, S-171 76 Stockholm, Sweden

## Abstract

The low molecular weight compound, PRIMA-1^MET ^restores the transcriptional transactivation function of certain p53 mutants in tumor cells. We have previously shown that PRIMA-1^MET ^induces nucleolar translocation of p53, PML, CBP and Hsp70. The Epstein-Barr virus encoded, latency associated antigen EBNA-5 (also known as EBNA-LP) is required for the efficient transformation of human B lymphocytes by EBV. EBNA-5 associates with p53-hMDM2-p14ARF complexes. EBNA-5 is a nuclear protein that translocates to the nucleolus upon heat shock or inhibition of proteasomes along with p53, hMDM2, Hsp70, PML and proteasome subunits. Here we show that PRIMA-1^MET ^induces the nucleolar translocation of EBNA-5 in EBV transformed B lymphoblasts and in transfected tumor cells. The PRIMA-1^MET ^induced translocation of EBNA-5 is not dependent on the presence of mutant p53. It also occurs in p53 null cells or in cells that express wild type p53. Both the native and the EGFP or DSRed conjugated EBNA-5 respond to PRIMA-1^MET ^treatment in the same way. Image analysis of DSRed-EBNA-5 expressing cells, using confocal fluorescence time-lapse microscopy showed that the nucleolar translocation requires several hours to complete. FRAP (fluorescence recovery after photobleaching) and FLIP (fluorescence loss in photobleaching) measurements on live cells showed that the nucleolar translocation was accompanied by the formation of EBNA-5 aggregates. The process is reversible since the aggregates are dissolved upon removal of PRIMA-1^MET^. Our results suggest that mutant p53 is not the sole target of PRIMA-1^MET^. We propose that PRIMA-1^MET ^may reversibly inhibit cellular chaperons that prevent the aggregation of misfolded proteins, and that EBNA-5 may serve as a surrogate drug target for elucidating the precise molecular action of PRIMA-1^MET^.

## Introduction

Epstein-Barr virus (EBV) is the most powerful transforming agent of human cells. EBV infected resting B cells undergo blast transformation and develop into immortal lymphoblastoid cell lines (LCLs) *in vitro *or into immunoblastic lymphomas in immunocompromised host. Nine latent viral proteins are expressed regularly in the transformed cells: the nuclear antigens, EBNA1–6, and three membrane proteins, LMP-1, -2a and -2b. Six of these proteins are required for immortalization: EBNA-1, -2, -3 (3A), -5 (LP), -6 (3C) and LMP-1 [1,2]

EBNA-5 and EBNA-2 are the first two viral proteins expressed in newly infected cells [3]. The co-expression of EBNA-2 and EBNA-5 plays an important role already at the first steps of EBV induced transformation by driving resting B cells into the cell cycle [4]. The co-operation between EBNA-2 and EBNA-5 is needed for the activation of LMP-1 and Cp viral promoters [5].

EBNA-5 preferentially accumulates in distinct nuclear foci in EBV-transformed lymphoblastoid cell lines. We have previously shown that these foci are inside the PML bodies [6]. We have also shown that the same foci contained the retinoblastoma (Rb) protein and the heat shock protein, Hsp70 [7]. Artificial spreading of the chromatin by exposure to the forces of fluid surface tension disrupted the co-localization gradually. It showed that EBNA-5 is located in the inner core of the bodies whereas PML formed the rigid outer shell [6].

Nuclear bodies with prominent PML staining are seen in uninfected resting B lymphocytes. This staining pattern does not change upon EBV infection. In freshly infected cells EBNA-5 is diffusely distributed throughout the nucleoplasm but after a few days gradually relocate to the PML bodies and remains there in established lymphoblastoid cell lines [8]. The localization of EBNA-5 to PML bodies is restricted to EBV- infected human lymphoblasts. Exogenously expressed EBNA-5 in any other cell lines distributes homogeneously in the nucleoplasm [9].

Heat shock or high cell density induced metabolic stress leads to the translocation of EBNA-5 to the nucleoli both from the PML bodies or from the nucleoplasm [10]. Subsequently we found that proteasome inhibitors induced also nucleolar translocation of EBNA-5 in both LCLs and transfected cell lines. They also induced nucleolar translocation of Hsp70 protein and mutant p53. Translocation of the later was enhanced by the presence of EBNA-5 [9]. In a separate study, we have shown that proteasome inhibitors can induce nucleolar translocation of various components of the PML body and nuclear/nucleolar accumulation of proteasomes [11].

The precise role of EBNA-5 in the virus induced transformation has nor yet been established. It binds to p14ARF-hMDM2-p53 complexes. Forced overexpression of p14ARF leads to the formation of extranucleolar inclusions with subsequent entrapment of hMDM2, p53 and EBNA-5 [6,12-14].

PRIMA-1 has been identified through differential screening of the structural diversity set of the NCI chemical library, as a drug that selectively induces apoptosis in mutant p53 bearing human tumor cells but not in their p53 -/- counterparts [15]. Treatment with PRIMA-1 suppresses the growth of mutant p53 expressing human tumor xenografts *in vivo *[16,17]. PRIMA-1 can restore the transcriptional transactivating function of certain p53 mutants and induce p53 dependent apoptosis [15].

Recently, we have shown that PRIMA-1^MET^, a methylated derivative of the original PRIMA with improved pro-apoptotic activity, causes nucleolar translocation of mutant p53 and of PML, CBP and Hsp70. The level of Hsp70 was significantly increased by PRIMA-1^MET ^treatment. The nucleolar accumulation of PML, CBP and Hsp70 was much more efficient in cells with mutant p53 as compared to p53-/- cells [18]. PRIMA-Dead, a compound structurally related to PRIMA-1^MET ^but unable to induce mutant p53-dependent apoptosis, failed to induce nucleolar translocation of mutant p53. These results suggested that redistribution of mutant p53 to nucleoli plays a role in PRIMA-1^MET ^induced apoptosis.

Considering the intimate relationship between EBNA-5 and the p53 pathway as well as the obvious similarities between the proteasome inhibitor and PRIMA-1^MET ^induced nucleolar translocation of p53, PML and Hsp70, we have now investigated the effect of PRIMA-1^MET ^on the subcellular distribution of EBNA-5. Here we show that PRIMA-1^MET ^induces the nucleolar translocation of both virus encoded endogenous and transfected exogenous EBNA5 and its fluorescent derivatives GFP-EBNA5 and DSRed-EBNA-5.

## Methods

### Cell cultures

All cell lines were grown in Iscove's cell culture medium supplemented with 10% heat-inactivated FBS, 2 mM L-glutamine, 100 U/ml penicillin and 100 U/ml streptomycin. The cells were passaged every fourth day 1:5. Cultures were regularly tested for the absence of mycoplasma with Hoechst 33258 staining. Transfections were done using Lipofectamine Plus reagent (GibcoBRL) according to the manufacturer's instructions. In the present study the following cell lines were used: LSsp, EBV transformed lymphoblastoid B-cell line, H1299 lung adeno carcinoma line (p53 -/-) and its mutant p53 transfected subline (with His 175), SW480, a colorectal cancer line with mutant p53 (Arg to His 273 and Pro to Ser 309). MCF-7, a breast carcinoma line bearing wild-type p53. Clones of MCF-7 and SW480 lines (S2 carrying EBNA-5 and P2 vector control), constitutively expressing EBNA-5 from a pBabe-EBNA-5 construct were generated using selection with 1 μg/ml puromycin (Sigma). MCF7 cells constitutively expressing DsRed_EBNA-5 were selected on 1 mg/ml G418 (Sigma).

### Constructs

GFP-EBNA-5 was made by cloning an EBNA-5-encoding *Bam*HI-*Eco*RI fragment from pBabe-EBNA-5, containing four W repeats and the unique C-terminal region, into *Bgl*II-*Eco*RI-cleaved pEGFP-N1 [13]. To produce red fusion protein EBNA-5 was amplified with primers containing NheI-EcoRI 5'overhangs. The cleaved PCR product was cloned into the corresponding sites of pDsRed1-N1 vector (Clontech).

### PRIMA-1^MET^-treatment and immunofluorescence staining

The monoclonal mouse antibody JF186 was used against EBNA-5 [19] and the MAb used against B23/nucleophosmin was a gift from P. K. Chan, Baylor College of Medicine, Houston, USA. The cells were cultured on cover slips in six-well plates until they have reached a density of 5 × 10^4 ^cells/cm^2 ^and incubated for 24 h in the presence of 50 μM PRIMA-1^MET ^dissolved in dimethylsulphoxide (DMSO). Cells treated with DMSO were used as controls.

Cells were fixed with methanol-acetone (1:1) at -20°C and then were re-hydrated in phosphate-buffered saline (PBS) for 30 min. Antibodies were diluted in blocking buffer (2% bovine serum albumin, 0.2% Tween-20, 10% glycerol in PBS). The cells were stained with primary MAbs for 1 h at room temperature, followed by three washes in PBS, incubated with secondary FITC or Texas red-conjugated antibodies, washed three times and mounted with 80% glycerol solution in PBS containing 2·5% 1,4-diazabicyclo-(2.2.2)octane (Sigma). Bisbenzimide (Hoechst 33258) was added at a concentration of 0·4 μg/ml to the secondary antibody for DNA staining.

### Live cell microscopy

Long term live cell fluorescence imaging was carried out in POC Mini chambers. The DSRed-EBNA-5 expressing cells were grown on the round cover glass insert of the POC Mini chamber (Carl Zeiss), The chambers were loaded with 400 ul cell culture medium and were used in fully closed mode. The imaging was carried out on an Ultraview LCI three line laser Nipkow spinning disc confocal microscope system with a CSU10 Yokogawa head (Perkin Elmer) assembled on a Zeiss Axiovert motorized microscope. During the imaging the temperature was maintained at 37 C with the help of heat controller block covered with a transparent plexi shield. The heat loss, toward the oil immersion objective, was prevented by the use of a separate objective heather ring. Both the controller block and the heater ring were controlled by a common electric thermostate. The four dimensional image capture was carried out using the custom developed software "FiveColorMovie" written by us, using the Openlab Automator visual programming environment (Improvision). To ensure the maintenance of proper focal plane during the extended imaging periods we have developed an autofocusing program module that uses recursive minimum/maximum intensity measurements at 100 separate small squares of a matrix overlayed on the gaussian blurred cental region of interest (ROI) of the raw image. To find the focus, series of short (50 microsecond) expositions are made at different Z axis positions that are clustered around the last used focal plane, and extended in height that is twice the length of the imaging stack. The section with the greatest difference between the minimum and maximum intensity values is selected as sharpest. The Z position of the sharpest image then serves as the central point for the consequent capturing of 10 images arranged in a Z stack with exposition times in the range of 500 to 1000 milliseconds. To conveniently capture several hundreds of multicolour images along the time axis the individual Z stacks were collapsed "on-the-fly" using maximum intensity projection algorithm. By saving the images after every 200 time points the program assures that the image capture process is not limited by the RAM but by the available hard disc space.

### Photokinetic measurements of recombinant EBNA-5 in live cells

To measure the intracellular mobility of EBNA-5 in different intranuclear compartments of treated and control cells we used an Ultraview RS five line laser Nipkow spinning disc confocal system with a CSU22 Yokogawa head (Perkin Elmer) assembled on a Nikon inverted fluorescence microscope. To achieve pixel precise bleaching of selected areas a galvanometric Photokinesis unit (Perkin Elmer) with separate laser input was installed between the Ultraview unit and the photoport of the microscope. The timelapse 4D imaging with single (FRAP) and repeated (FLIP) bleach cycles was carried out with the Ultraview capture software (Perkin Elmer). The captured images were quantified using the analytic routines of the Ultraview program as well as the program ImageJ (Rasband, W.S., ImageJ, U. S. National Institutes of Health, Bethesda, Maryland, USA, ). For FRAP and FLIP studies DSRed-EBNA-5 expressing cells were grown on the bottom glass of a POC Mini chamber. The selected areas were bleached using the 568 nm line of an Argon-Krypton laser. In a typical FRAP recording two prebleach images were followed by 100 bleach cycles (total 300–1000 ms) and the recovery was measured by a series of 500 ms exposition over 1 to 3 minutes (120–360 images). In the FLIP experiments the bleach cycles were followed by the capture of ten images and then the bleach cycles were repeated several times.

## Results

### Immunofluorescence staining of EBNA-5 in fixed cells

To study the effect of PRIMA-1^MET ^on the subcellular localization of EBNA-5, EBV transformed lymphoblastoid cell lines and EBNA-5 transfected tumor cells were treated with various concentrations of the drug for 24 hours. Beside native EBNA-5 we have also used EBNA-5 conjugated to the C-terminus of the fluorescence protein EGFP or DSRed. Upon completion of the treatment the cells were fixed with methanol:aceton and stained with the monoclonal anti-EBNA-5 antibody JF186.

**Figure 1:**
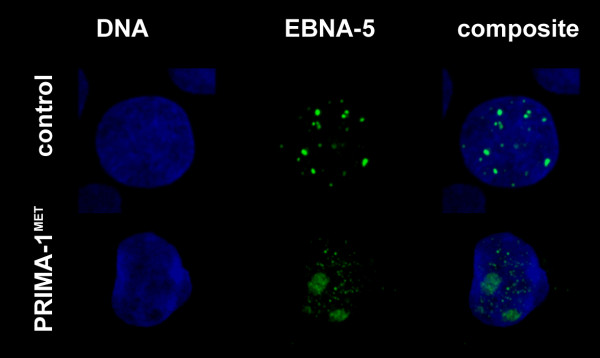
**PRIMA-1^MET^-induced translocation of virus encoded endogeneous EBNA-5 in the Epstein-Barr virus transformed human lymphoblastoid cell line LSsp that carries virus encoded EBNA-5 and harbors wild type p53**. 24 hours treatment with 50 uM PRIMA-1^MET ^leads to the disappearance of EBNA-5 from the PML bodies and to relocation to the nucleolus.

**Figure 2:**
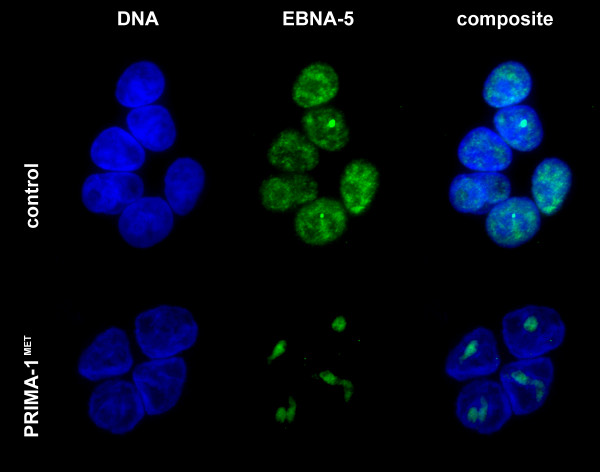
**PRIMA-1^MET^-induced translocation of exogenous EBNA-5 in mutant p53 carrying SW480 colon carcinoma cells after 24 hours treatment**. Immunofluorescence staining of EBNA-5 is green, DNA staining using Hoecsht 33258 is blue.

We found that 12–24 hours treatment with 50 uM PRIMA-1^MET ^induced nucleolar translocation of EBNA-5 in all cell lines such as the lymphoblastoid cells LSsp expressing virus encoded endogenous EBNA-5 (Figure 1) and transfected tumor cell lines such as the colon carcinoma line SW480 (endogenous mutant p53; Figure 2), the breast carcinoma line MCF7 (wt p53) and the lung adeno-carcinoma line H1299 (p53 null cells) as well as its transfected variant expressing mutant p53 (His175). The identity of the nucleolus was ascertained by B23 staining and/or phasecontrast imaging in parallel.

**Figure 3:**
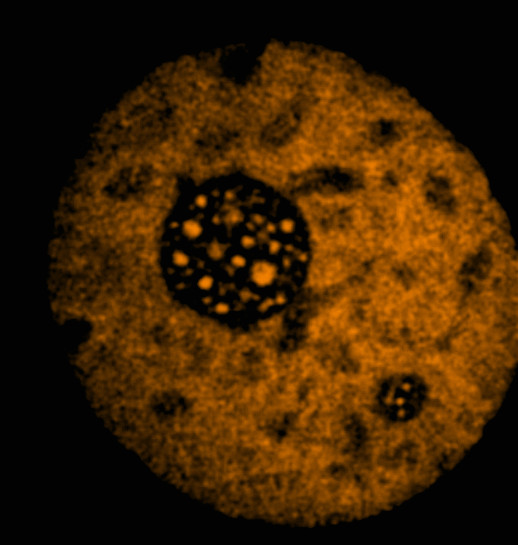
**Distribution of DSRed-EBNA-5 in the nucleus of a living MCF7 cell that carries wild type p53**. Single confocal section selected from the middle of a series of 21 images.

In untreated, transfected cells EBNA-5 localized to the low DNA density areas corresponding to the euchromatin (Figure 3). It was either absent from the nucleolus or if present its level did not exceed the amount in the nucleoplasm (Additional file 1 and additional file 2). In the treated cells there was an overall increase of EBNA-5 levels with a very prominent increase in the nucleolus. After 20 hours of treatment almost all diffuse nucleoplasmic EBNA-5 signal was concentrated in numerous distinct foci evenly distributed throughout the entire nucleus.

To test the possible influence of EBNA-5 on the survival rate of PRIMA-1^MET ^treated cells we have compared wild type and mutant p53 carrying cells stably transfected with EBNA-5 and their vector controls. PRIMA-1^MET ^treatment had no effect on the survival rate of wild type p53 carrying MCF7 breast carcinoma cells with or without EBNA-5 at any PRIMA-1^MET ^concentrations, although the cells showed prominent nucleolar translocation of EBNA-5. Comparing EBNA5 transfected (S2) and vector control transfected (P2) SW480 colon carcinoma cells that express mutant P53 we found that the presence of EBNA-5 slightly sensitized to the PRIMA-1^MET ^effect. (Additional file 3).

### Kinetics of nucleolar translocation of DS-redEBNA5 cells in PRIMA-1MET treated cells

**Figure 4:**
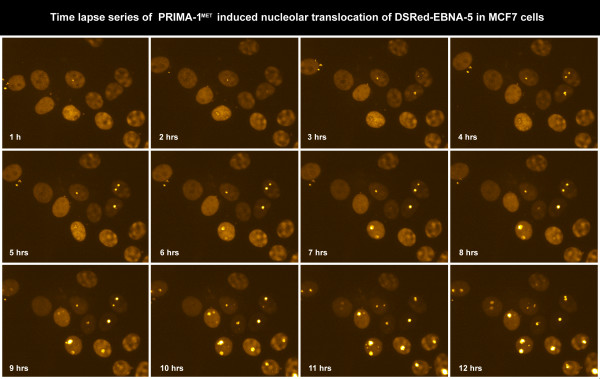
**Time lapse series of PRIMA-1^MET^-induced translocation of DSRed-EBNA-5 in stably transfected MCF7 cells**. Images at 1 hour interval were selected from a series of 720 images recorded in every minutes for 12 hours.

**Figure 5:**
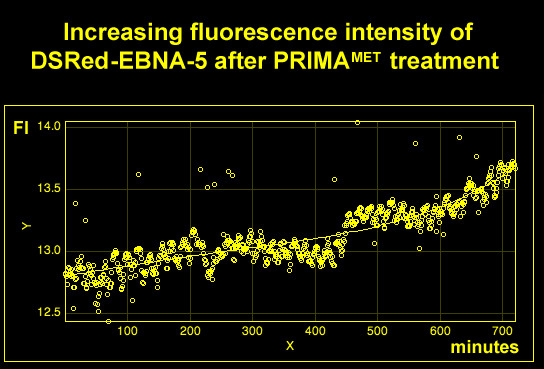
**PRIMA-1^MET ^treatment leads to an overall increase of DSRed-EBNA-5 levels as shown by plotting the average fluorescence intensity against time of the 720 frames shown in Figure 4 after background subtraction**.

To study the dynamics of PRIMA-1^MET ^induced nuclear movements of EBNA-5 we used an automated confocal microscopy method, developed by us, for live cell imaging (see Materials and Methods). The technique permits continuous recording of living cells using a combination of fluorescence and phase contrast illumination over several (6–24) hours. We found that the cells tolerated excellently the combination of 568 nm epifluorescence and 600 nm transmitted phase contrast illumination. On the other hand using shorter wavelengths (405 and 488 nm) always led to visible phototoxic effects during prolonged experiments. Imaging cells stably transfected with DSRed-EBNA-5 revealed that EBNA-5 that was originally evenly distributed in the nucleoplasm successively accumulated in the nucleoli between 6 and 10 hours after the PRIMA-1^MET ^treatment (Figure 4 and Additional file 4). EBNA-5 accumulation was associated with the formation of 15–20 round or ovoid particles of the size of 250–300 nm that showed limited movement inside the nucleolus. The nucleolar accumulation has regularly started from a single focus in a given nucleolus. Different nucleoli started the process at different time. Some nucleoli showed up to four hours delay as compared to the earliest accumulating nucleoli in the same nucleus. After 10 hours treatment with PRIMA-1^MET ^the nucleoli became saturated with EBNA-5. At this point some of the brightly fluorescent DSRed-EBNA-5 particles were released from the nucleolus and moved around in the nucleoplasm by rapid Brownian movement (Additional file 5). The nucleolar accumulation was also accompanied by an overall increase of DSRed-EBNA-5 fluorescence intensity (Figure 5).

### Effect of PRIMA-1^MET ^on the mobility of DSRed-EBNA-5

We carried out FRAP and FLIP analysis on untreated and PRIMA-1^MET^-treated, DSRed-EBNA-5 transfected MCF7 cells to measure the rate of mobility of EBNA-5 in different nuclear sub-compartments. In the untreated control cells the bulk of DSRed-EBNA-5 was homogenously distributed throughout the nucleoplasm. This fraction showed very high mobility. Using single bleaching FRAP with 2 um spot size the average half recovery time (T 1/2) was 1.5 second that corresponds to a diffusion coefficient of 0.66 um^2^/s. In comparison the calculated mobility in free solution would be 54.6 um^2^/s.

**Figure 6:**
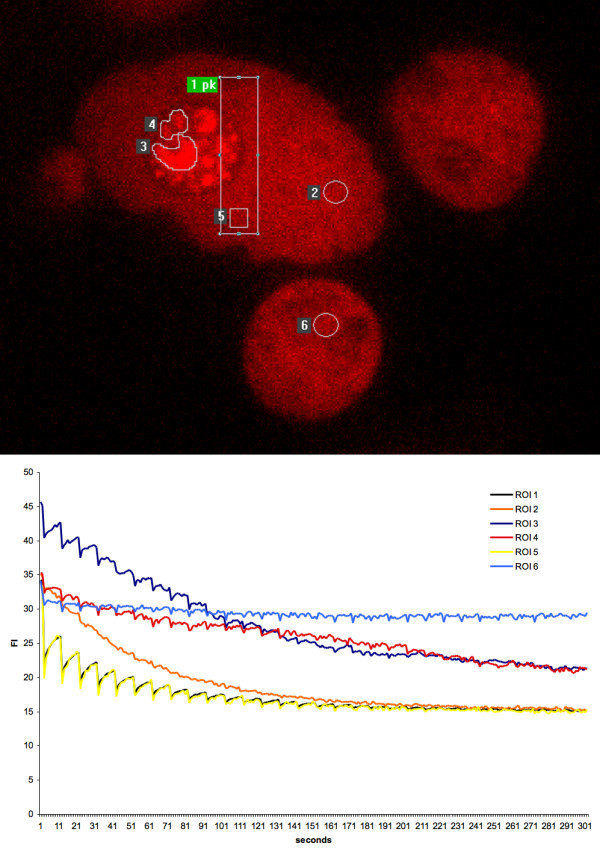
**Fluorescence loss in photobleaching (FLIP) experiment of untreated DSRed-EBNA-5 expressing MCF7 cells show very high mobility of homogeneously distributed DSRed-EBNA5**. Region of interest 1 (ROI 1) was bleached for 300 ms followed by recording of 10 consecutive images at 500 ms intervals. These cycles were repeated for 5 minutes. ROI 1 and 5, representing the total bleached area and a selected bleached subarea, showed rapid recovery of fluorescence. ROI 2, representing a remote non-bleached area in the nucleoplasm showed rapid homogeneous decrease of fluorescence. ROI 6 in adjacent cell showed no changes. ROI 3 and 4 in the nucleolus show two differently equilibrating compartment one faster (ROI 3) and one slower (ROI 4).

Imaging the fluorescence loss in the non-illuminated areas (FLIP) showed a rapid depletion of the homogeneous signal from the entire nucleoplasm. The minor population of nucleolar DSRed-EBNA-5 in the non-treated cells showed a higher resistance to FLIP. The nucleolar DSRed-EBNA-5 was localized to distinct separated areas inside the nucleolus. Using pixel precise bleaching of the individual areas in FRAP experiments we could identify two levels of recovery one intermediate (T 1/2 21 second) and one very slow (T 1/2 >> 300 s). The presence of the two separate nucleolar subcompartment was also confirmed by FLIP experiments. (Figure 6 and Additional file 6)

**Figure 7:**
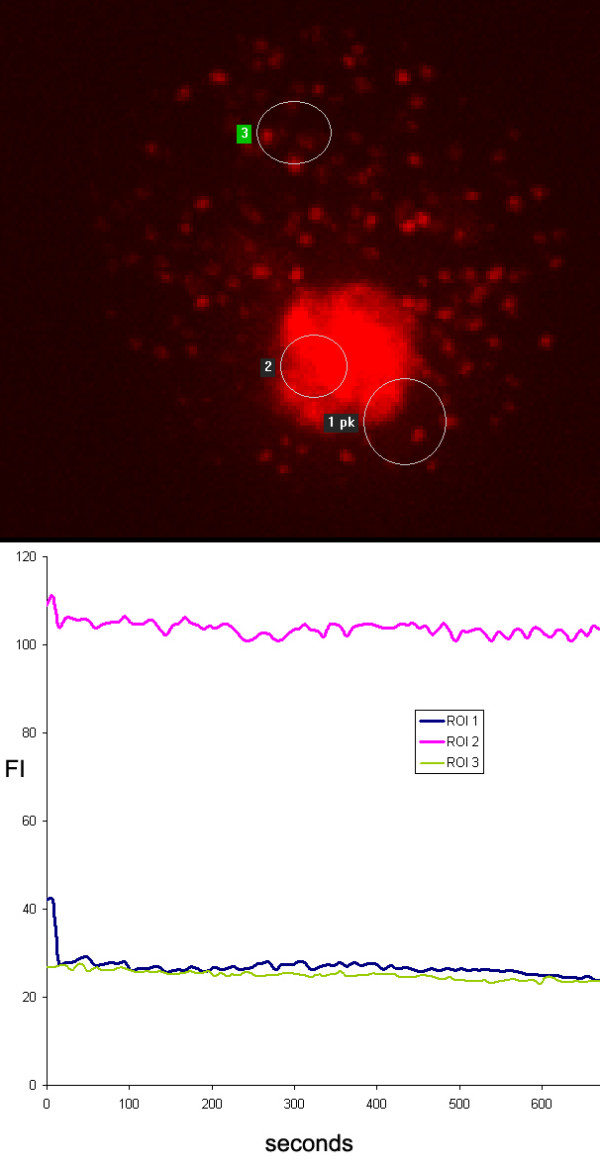
**20 hours treatment with PRIMA-1^MET ^leads to the loss of mobility of DSRed-EBNA-5 in MCF7 cells as shown by FLIP experiment**. ROI 1 was bleached as above but showed no significant recovery. Adjacent ROI 2 and distant ROI 3 showed no significant loss of fluorescence.

Treatment with PRIMA-1^MET ^led to the accumulation of DSRed-EBNA-5 in well-defined granules in the nucleolus with very low molecular mobility. In other words, repeated bleaching of adjacent nucleoplasmic areas, separate DSRed-EBNA-5 positive foci in the same nucleolus or even part of the same granules failed to induce any significant loss of fluorescence in the non-bleached structures (Figure 7).

After 20 hours of treatment DSRed-EBNA-5 in addition to the nucleolar aggregates formed rigid bodies evenly scattered in the nucleoplasm. FLIP showed that these bodies contained DSRed-EBNA-5 with similar low exchange mobility as the nucleolar aggregates. These bodies showed also relatively rapid translational Brownian movement similarly to the ones released from overfilled nucleoli. An important distinction however from the former was that the nucleoplasmic bodies were somewhat larger (400 nm versus 300 nm) and their movement trajectories were much more restricted (Figure 7 and Additional file 7). Whereas the bodies released from the nucleolus could travel to any area of euchromatin the bodies that were precipitated out at the later time point in the nucleoplasm moved within a well-defined sphere of 800–1200 nm. After 24 hours of treatment the nucleoplasmic bodies increased in size but not in number (Additional file 8).

### Reversibility of PRIMA-1^MET ^induced aggregation of DSRed-EBNA-5

**Figure 8:**
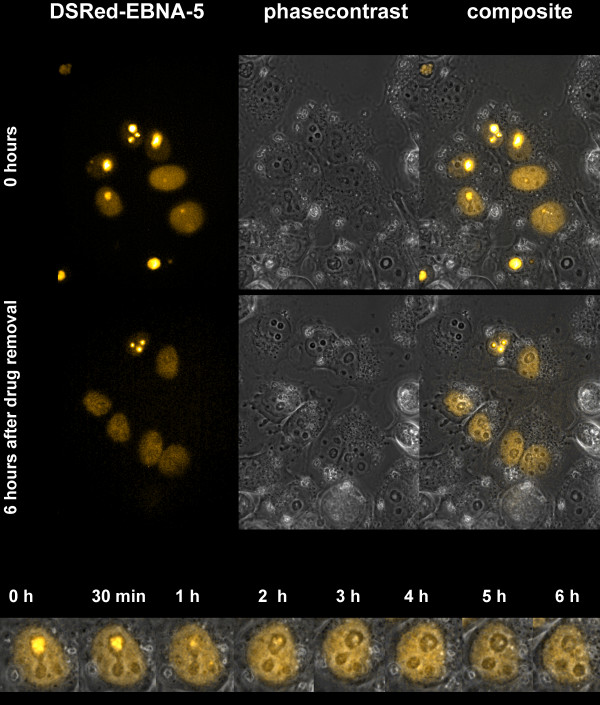
**The PRIMA-1^MET^-induced nucleolar translocation of DSRed-EBNA-5 is reversible in the p53 -/- H1299 cells**. The translocation was induced by 12 hours drug treatment followed by extensive washing with drug free medium. Time-lapse movie was recorded for 6 hours, one frame per minute. The fluorescence images are combined with phase contrast pictures to demonstrate the intact cellular morphology of the cells during the entire length of the experiment.

PRIMA-1^MET ^regularly induces apoptosis in mutant p53 expressing cells. To explore the possibility whether the protein aggregation phenomenon is a feature advanced stage cellular agony we have treated DSRed-EBNA-5 expressing, p53 -/-, H1299 cells with PRIMA-1^MET ^for 12 hours. These cells are much less sensitive to PRIMA-1^MET ^induced apoptosis than its mutant p53 expressing derivatives. The drug treatment induced nucleolar accumulation of the protein in most nucleoli. Removing PRIMA-1^MET ^by repeated washing with drug free medium led to the complete dissolution of the aggregates as it could be demonstrated by combined fluorescent/phase contrast time lapse microscopy. No cytopathic effects were detected at any time during the experiment (Figure 8).

## Discussion

The most straightforward explanation of the observed effects is that PRIMA-1^MET ^induces a gradual precipitation of EBNA-5. In untreated cells EBNA-5 moves around with high mobility in the nucleoplasm but slows down in the nucleolus. We suggest that this is due to the mechanical sieving effect of the densely arranged chromatin fibers in the *zona fibrillaris *of the nucleolus (see Additional file 2 for illustration of the density of chromatin fibers in the nucleoplasm and in the nucleolus in a living cell nucleus). In our scenario the drug treatment induces gradual aggregation of EBNA-5 and the aggregates start to clog the nucleolar chromatin fiber meshwork. Heterogeneity in the sieving effect ("effective pore size") of different subnucleolar compartments or even between different nucleoli could explain the phenomena of: a.) focal initiation of nucleolar accumulation b.) nucleolar subcompartments with different mobility identified by FLIP c.) asynchronous accumulation of EBNA-5 in different nucleoli.

Along the same lines we also suggest that upon prolonged (20+ hours) treatment with PRIMA-1^MET^, the in situ falling out of EBNA-5 precipitates in the nucleoplasm is due to the convergence between the size of the precipitated bodies and the sieving dimensions of the 300 nm chromatin fibre meshwork of the nucleoplasm. This is also consistent with the finding that nucleoplasmic EBNA-5 aggregates show spatially restricted and uniform random walk trajectories (Additional file 7 and Additional file 8).

Conformational change due to mutation or thermal, acid-base or redox change that leads to increased surface exposition of otherwise cryptic hydrophobic side chains is the most usual cause of protein precipitation. Exposed hydrophobic surfaces are potent inducers of cellular heat shock responses. Increased exposition of hydrophobic surfaces lead to increased expression of molecular chaperons that actively counteract the precipitation process, by isolating the misfolded proteins from each other and using the energy of hydrolyzed ATP for actively "massaging" their protein client back to a native conformation that represents the lowest level of folding energy. Importantly mutant p53 was recently found in complex with Hsp90 in PRIMA-1^MET ^treated cells [20]. Hsp90 is a major constituent of the proteome, comprising up to 5% of the total mass of cellular proteins. It remains to be elucidated to what extent mutant p53 is associated with other, less abundant heat shock proteins in the presence and absence of PRIMA-1^MET^.

It is well known however that mutant p53 often complexes with Hsp70 [21]. Association of Hsp70 and CHIP (carboxy terminus of Hsp70-interacting protein) is responsible for ubiquitination and degradation of some of the p53 mutants [22]. Our present findings suggest that PRIMA-1^MET ^might act through reversible inhibition of cellular chaperon activity. In this scenario the aggregation of EBNA-5 (or mutant p53) would be prevented by the constitutive activity of Hsp70 type cellular chaperons. Inhibition of chaperon activity by PRIMA-1^MET ^would lead to the accumulation of protein aggregates as well as a reactive increase of Hsp70 expression.

Direct interaction with the Hsp70 family of chaperons is also a common denominator of EBNA-5 and mutant p53. The Hsp70 family members are also among the most potent anti-apoptotic agents that can block both the extrinsic (membrane signalling initiated) and the intrinsic (mitochondrial cytochrome C release initiated) pro-apoptotic signal pathways [23].

Transformation of primary B-cells by EBV is dependent on the EBNA-2 induced activation of numerous viral and cellular genes [3,4,24,25]. EBNA-5 enhances this transactivation. Hsp70 is a major complexing partner of EBNA-5 [26]. Elevated expression of Hsp70 increases, and suppressed expression of Hsp70 decreases the effect of EBNA-5 on EBNA-2 mediated transactivation. It has been suggested that Hsp70 chaperon activity may help EBNA-5 in shuttling off repressors from EBNA2-enhanced promoters [27].

We have previously shown that inhibition of proteasome activity leads to synchronous nucleolar translocation of mutant p53, EBNA-5 and Hsp70 in transfected SW480 cells. We have also shown that increased level of EBNA-5 enhanced the nucleolar translocation of mutant p53 [9]. More recently we found that the simultaneous presence of EBNA-5 and mutant p53 sensitizes cells for MG132 and bortezomib induced cytotoxicity (R. Ötvös et al, to be published). These data also suggest there is a close functional interplay between mutant p53 and EBNA-5.

PRIMA-1^MET ^was identified as a low molecular weight compound that can enhance apoptosis in mutant p53 carrying cells, compared to the p53 null parental cells. Most p53 mutants are in complex with Hsp70 proteins. We have recently shown that PRIMA-1^MET ^treatment increases Hsp70 expression and nucleolar translocation, in parallel with the induction of nucleolar accumulation of mutant p53 [18]. Numerous experiments indicate that the increased apoptosis induction is accompanied by the activation of p53 target genes [28-32]. Several lines of evidence suggest that PRIMA-1^MET ^can also act independently of the p53 status of the cell. It can radiosensitize prostate carcinoma cell lines with mutant or wild type p53 and p53 -/- cells as well [33]. Introduction of mutant p53 (p53ser249 or p53gln248) into p53 -/- hepatocarcinoma cells increases sensitivity to PRIMA-1^MET ^without the induction of p53 target genes [34].

PRIMA-1^MET ^is a powerful apoptosis-inducing agent. Identification of its targets is partly hindered by the difficulties to separate its effect from the numerous molecular changes associated with cellular agony subsequent to drug treatment. Here we show that subcellular distribution of EBNA-5 changes in parallel with mutant p53 itself upon PRIMA-1^MET^treatment. Similar changes in EBNA5 distribution occur in cells that lack p53 and are therefore resistant for p53 induced apoptosis. We propose that EBNA-5 may serve as a surrogate target that may help to elucidate the molecular action of PRIMA-1^MET^.

## Competing interests

The authors declare that they have no competing interests.

## Authors' contributions

Laboratory work, transfection, cell culturing and immunostaining was carried out by GS, PRIMA-1^MET ^treatment was made by GS and NR, microscopic analysis and programming was performed by EF, GP and GS, viability assay upon MG132 and PRIMA-1^MET ^treatment was performed by RÖ, DS-redE5 plasmid and transfection of MCF7 cells with it was made by EK. The experimental plan was conceived by KW GK and LS and it was evaluated by GK and LS. All the authors have read the manuscript and agreed with its content.

## Supplementary Material

Additional file 1 **Distribution of DSRed-EBNA-5 in the nucleus of MCF7 cell (corresponding to Figure**3**). **In stably transfected cells DSRed-EBNA-5 is distributed homogeneously in the euchromatin and in well-defined round spaces in the zona granulosa of the nucleolus. Importantly the fluorescence intensity of the fusion protein is the same in the nucleolar and in the nucleoplasmic fraction. Left side – Animation moving through a series of 21 confocal sections along the Z axis. Rigth side – 3D projection and 360° rotation of the maximum intensity projected images around the Y axis. Click here for file

Additional file 2 **Six minutes time-lapse movie, showing DSRed-EBNA-5 in the nucleus of MCF7 cell.** Single confocal section with sampling in every 2 seconds (122 timepoints). The movie shows that in the nucleoplasm DSRed-EBNA-5 fills up the space between the bundles of 300 nm chromatin fibres. The fiber bundles appear as dark shadows over the uniformly bright fluorescence background. The film also shows that the chromatin fibers are more densely packed area that corresponds to the fibrillar compartment of the nucleolus thus providing the structural basis for the postulated molecular sieve with small pore size. Click here for file

Additional file 3 **Survival rate of PRIMA-1^MET ^treated MCF7 (wtp53) and SW480 (mp53) cells in the presence and absence of EBNA-5.** Click here for file

Additional file 4 **Time-lapse movie of PRIMA-1^MET^-induced nucleolar accumulation of DSRed-EBNA-5 in stably transfected MCF7 cells (corresponding to Figure**4**). **The nucleolar accumulation starts non-synchronously in different nuclei between 3–9 hours after exposition to the drug. Click here for file

Additional file 5 **High resolution magnified image of a single nucleus from the movie of Additional file**3**. **The movie shows that the accumulation of DSRed-EBNA-5 is starting non-synchronously in different nucleoli initiating from single foci. The movie also illustrates the escape of precipitated DSRed-EBNA-5 particles from the nucleolus and their travelling through the nucleoplasm by random Brownian movement. Click here for file

Additional file 6 **Time lapse movie of the FLIP experiment on non treated cells expressing DSRed-EBNA-5 as presented in Figure**6**. **Note the rapid loss of nucleoplasmic fluorescence in the non-bleached area of the targeted nucleus and the relative resistance of nucleolar fluorescence to FLIP effect, indicating the lower mobility DSRed-EBNA-5 in the nucleolar subcompartments. Click here for file

Additional file 7 **Time-lapse movie of the FLIP experiment on PRIMA-1^MET^- treated cell shown in Figure**7**. **The movie clearly shows that there is no loss of fluorescence even in the immediate neighbourhood of the bleached area indicating complete immobilisation (precipitation) of DSRed-EBNA-5 upon drug treatment. Also observe the fine granular precipitates in the nucleoplasm showing random Brownian movement restricted to the dimensions of the average distance between the nucleoplasmic chromatin fibers (as was illustrated in Additional file 2). Click here for file

Additional file 8 **Time lapse movie of treated cells 24 hours after the administration of PRIMA-1^MET^.** Note the pronounced increase in the size of nucleoplasmic granules. Click here for file
