# Supplementary figures and images for: PRIMA-1MET induces nucleolar translocation of Epstein-Barr virus-encoded EBNA-5 protein
Source: Mol Cancer. 2009 Mar 26;8:23. doi: 10.1186/1476-4598-8-23 (PMC2667484; doi:10.1186/1476-4598-8-23)

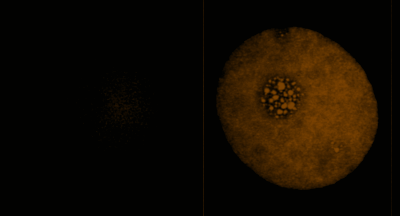

Supplement: Additional file 1 — Distribution of DSRed-EBNA-5 in the nucleus of MCF7 cell (corresponding to Figure3). In stably transfected cells DSRed-EBNA-5 is distributed homogeneously in the euchromatin and in well-defined round spaces in the zona granulosa of the nucleolus. Importantly the fluorescence intensity of the fusion protein is the same in the nucleolar and in the nucleoplasmic fraction. Left side – Animation moving through a series of 21 confocal sections along the Z axis. Rigth side – 3D projection and 360° rotation of the maximum intensity projected images around the Y axis. [file 1476-4598-8-23-S1.gif]

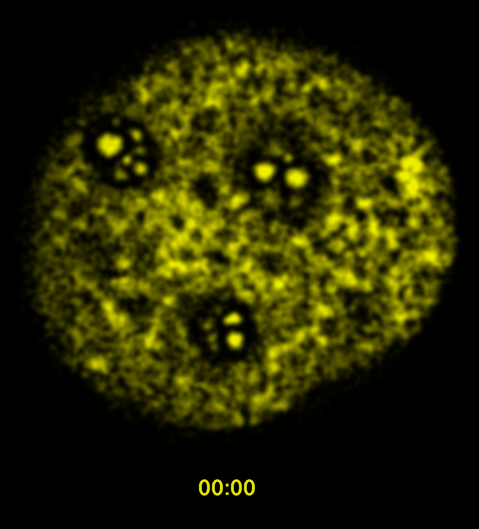

Supplement: Additional file 2 — Six minutes time-lapse movie, showing DSRed-EBNA-5 in the nucleus of MCF7 cell. Single confocal section with sampling in every 2 seconds (122 timepoints). The movie shows that in the nucleoplasm DSRed-EBNA-5 fills up the space between the bundles of 300 nm chromatin fibres. The fiber bundles appear as dark shadows over the uniformly bright fluorescence background. The film also shows that the chromatin fibers are more densely packed area that corresponds to the fibrillar compartment of the nucleolus thus providing the structural basis for the postulated molecular sieve with small pore size. [file 1476-4598-8-23-S2.gif]

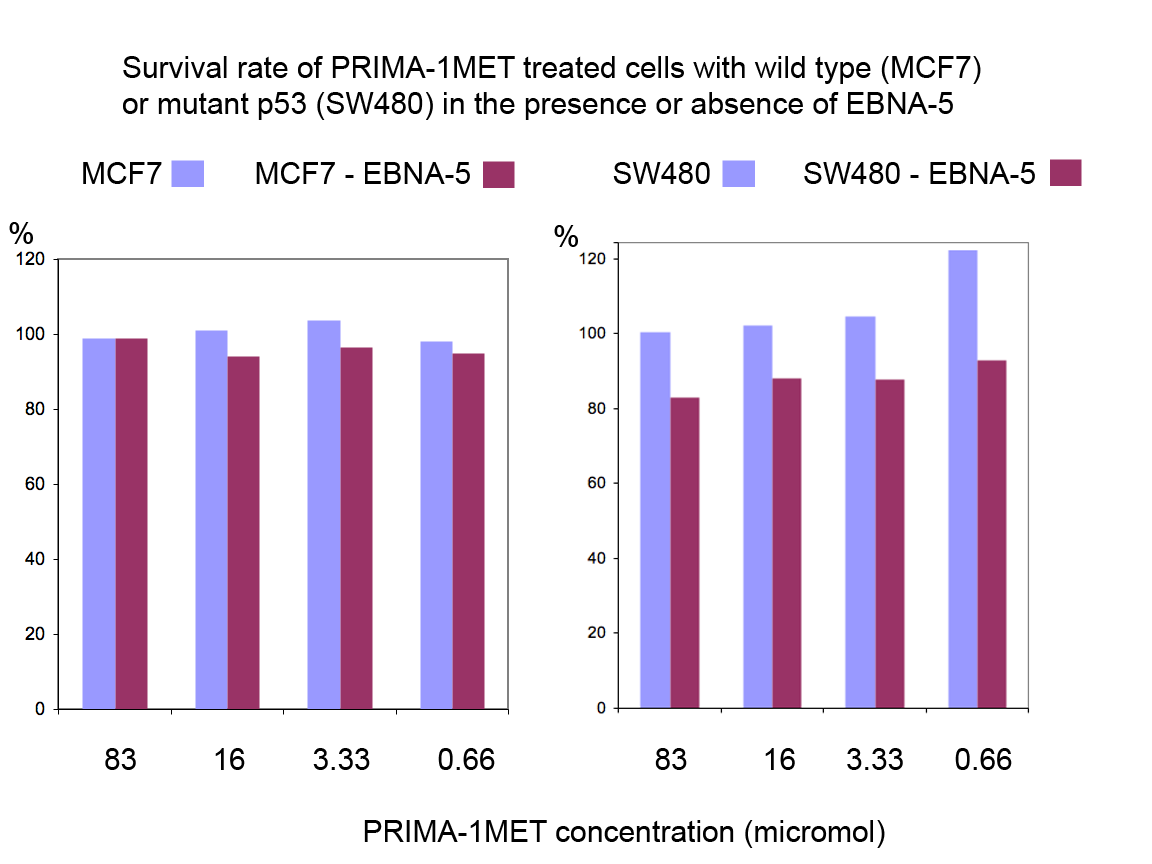

Supplement: Additional file 3 — Survival rate of PRIMA-1MET treated MCF7 (wtp53) and SW480 (mp53) cells in the presence and absence of EBNA-5. [file 1476-4598-8-23-S3.tiff]

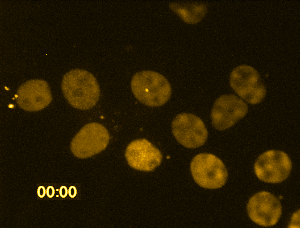

Supplement: Additional file 4 — Time-lapse movie of PRIMA-1MET-induced nucleolar accumulation of DSRed-EBNA-5 in stably transfected MCF7 cells (corresponding to Figure4). The nucleolar accumulation starts non-synchronously in different nuclei between 3–9 hours after exposition to the drug. [file 1476-4598-8-23-S4.gif]

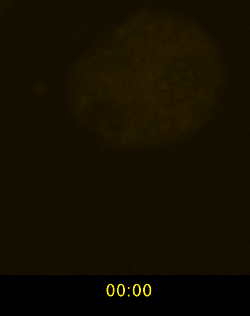

Supplement: Additional file 5 — High resolution magnified image of a single nucleus from the movie of Additional file3. The movie shows that the accumulation of DSRed-EBNA-5 is starting non-synchronously in different nucleoli initiating from single foci. The movie also illustrates the escape of precipitated DSRed-EBNA-5 particles from the nucleolus and their travelling through the nucleoplasm by random Brownian movement. [file 1476-4598-8-23-S5.gif]

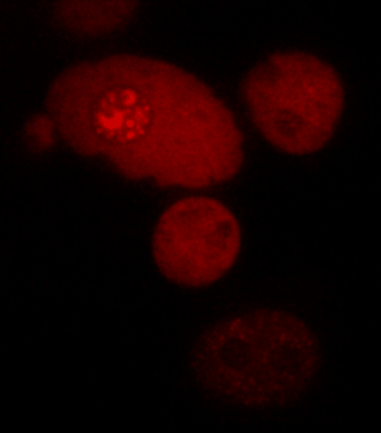

Supplement: Additional file 6 — Time lapse movie of the FLIP experiment on non treated cells expressing DSRed-EBNA-5 as presented in Figure6. Note the rapid loss of nucleoplasmic fluorescence in the non-bleached area of the targeted nucleus and the relative resistance of nucleolar fluorescence to FLIP effect, indicating the lower mobility DSRed-EBNA-5 in the nucleolar subcompartments. [file 1476-4598-8-23-S6.gif]

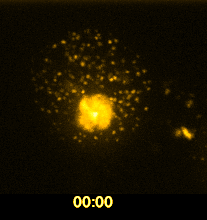

Supplement: Additional file 7 — Time-lapse movie of the FLIP experiment on PRIMA-1MET- treated cell shown in Figure7. The movie clearly shows that there is no loss of fluorescence even in the immediate neighbourhood of the bleached area indicating complete immobilisation (precipitation) of DSRed-EBNA-5 upon drug treatment. Also observe the fine granular precipitates in the nucleoplasm showing random Brownian movement restricted to the dimensions of the average distance between the nucleoplasmic chromatin fibers (as was illustrated in Additional file 2). [file 1476-4598-8-23-S7.gif]

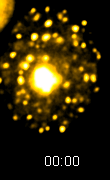

Supplement: Additional file 8 — Time lapse movie of treated cells 24 hours after the administration of PRIMA-1MET. Note the pronounced increase in the size of nucleoplasmic granules. [file 1476-4598-8-23-S8.gif]
